# Effects of brain–Computer interface combined with mindfulness therapy on rehabilitation of hemiplegic patients with stroke: a randomized controlled trial

**DOI:** 10.3389/fpsyg.2023.1241081

**Published:** 2023-10-09

**Authors:** Pei Wang, Jinyu Liu, Lili Wang, Huifang Ma, Xingyan Mei, Aihua Zhang

**Affiliations:** ^1^Shandong Provincial Hospital Affiliated to Shandong First Medical University, Ji’nan, Shandong, China; ^2^School of Nursing, Shandong First Medical University and Shandong Academy of Medical Sciences, Taian, Shandong, China; ^3^Linyi People’s Hospital, Linyi, Shandong, China

**Keywords:** stroke, brain-computer interface, mindfulness therapy, limb movement function, quality of life

## Abstract

**Aim:**

To explore the effects of brain–computer interface training combined with mindfulness therapy on Hemiplegic Patients with Stroke.

**Background:**

The prevention and treatment of stroke still faces great challenges. Maximizing the improvement of patients’ ability to perform activities of daily living, limb motor function, and reducing anxiety, depression, and other social and psychological problems to improve patients’ overall quality of life is the focus and difficulty of clinical rehabilitation work.

**Methods:**

Patients were recruited from December 2021 to November 2022, and assigned to either the intervention or control group following a simple randomization procedure (computer-generated random numbers). Both groups received conventional rehabilitation treatment, while patients in the intervention group additionally received brain–computer interface training and mindfulness therapy. The continuous treatment duration was 5 days per week for 8 weeks. Limb motor function, activities of daily living, mindfulness attention awareness level, sleep quality, and quality of life of the patients were measured (in T0, T1, and T2). Generalized estimated equation (GEE) were used to evaluate the effects. The trial was registered with the Chinese Clinical Trial Registry (ChiCTR2300070382).

**Results:**

A total of 128 participants were randomized and 64 each were assigned to the intervention and control groups (of these, eight patients were lost to follow-up). At 6 months, compared with the control group, intervention group showed statistically significant improvements in limb motor function, mindful attention awareness, activities of daily living, sleep quality, and quality of life.

**Conclusion:**

Brain–computer interface combined with mindfulness therapy training can improve limb motor function, activities of daily living, mindful attention awareness, sleep quality, and quality of life in hemiplegic patients with stroke.

**Impact:**

This study provides valuable insights into post-stroke care. It may help improve the effect of rehabilitation nursing to improve the comprehensive ability and quality of life of patients after stroke.

**Clinical review registration:**

https://www.chictr.org.cn/, identifier ChiCTR2300070382.

## 1. Introduction

Stroke is the second leading cause of death and the third leading cause of disability worldwide today (Owolabi et al., 2022). The Global Burden of Disease study (Feigin et al., 2021) shows the overall prevalence rate of stroke in China is approximately 39.9%, ranking first in the world, which means that approximately 2 out of every 5 people in a lifetime suffer from stroke in China. With the rapid growth in the proportion of aging population and urbanization, cardiovascular risk factors are also increasing, and with it the burden of stroke; additionally, there is a rapid increase in the proportion of low-income groups, obvious sex and regional differences, and the trend of younger age of onset is obvious (Perera et al., 2022). Despite improvements in survival rates, most stroke survivors often suffer from motor dysfunction, anxiety and depression, sleep disorders, cognitive disorders, and other complications (Rost et al., 2022). Nerve cell ischemia and hypoxia and muscle spasm caused by stroke are important causes of motor dysfunction after stroke, most patients have symptoms such as ataxia, limb pain and joint stiffness, which is one of the most common complications after stroke, and most patients are unable to perform self-care owing to disability (Malik et al., 2022). Furthermore, the incidence of post-stroke depression and sleep disorders is reported to be approximately 30% and 46% in patients with stroke (Baillieul et al., 2022; Guo et al., 2022), which significantly increases the risk of recurrent stroke. Therefore, the focus and difficulty of clinical rehabilitation is to enhance patients’ overall quality of life and health.

Studies have shown that exercise rehabilitation in patients post stroke is related to a good psychological and emotional status. Patients receiving post-stroke depression treatment demonstrate better motor and cognitive rehabilitation results, improved motor function, and reduction of negative emotions (Fotakopoulos and Kotlia, 2018; Saunders et al., 2020). Traditional rehabilitation therapies, such as intensive exercise training, restraint-induced exercise therapy, and neuromuscular electrical stimulation, have been reported to have different rehabilitation effects owing to their limitations; however, the combined efficacy of physical and mental rehabilitation has been hitherto ignored (Gittler and Davis, 2018).

In recent years, brain–computer interfaces (BCI) as a promising tool for motor function recovery provide a window for the real-time decoding of brain dynamics, facilitating external environment interaction through control signals derived from brain activity (Chaudhary et al., 2021). Within the context of motor rehabilitation, BCI systems use non-invasive electroencephalograms (EEG) to collect signals from patients and decode the intention of patients to move their affected limbs; therefore, a physical device can stimulate the paralyzed limb for early active rehabilitation training. Currently, a large amount of clinical evidence shows that BCI may be as efficacious as many traditional interventions for upper limb motor function rehabilitation after stroke (Cervera et al., 2018). The theory behind using EEG to analyze motor intention is that unilateral limb movement or imagined movement can stimulate the primary sensorimotor cortex and promote event-related desynchronization (ERD) on the opposite side of the brain. It is apparent in EEG data as a decline in the amplitude of an individual frequency’s rhythmic activity. The same side causes event-related synchronization (ERS), which causes an amplitude increase at a specific frequency (Pfurtscheller et al., 2000). Individuals vary greatly in capacity to perform motor imagination tasks, and each person has a unique combination of features. Research shows that the regions involved in Ml are remarkably similar to those involved in actual exercise, including the premotor conex (PMC), the primary motor area (M1), and the basal ganglia (BG), supplementary motor area (SMA), parietal cortex, cerebellum, cingulate gyrus, etc., (Hardwick et al., 2018). Neurophysiological research shows that better brain engagement significantly enhances activation of brain areas involved in motor rehabilitation, and that this activation could work as a compensatory link that makes up for the reduced activity of these brain regions caused by neurological disease (Danzl et al., 2012). Attention-driven brain activity increases large brain plasticity, which is crucial for successful rehabilitation (Bright et al., 2015).

Mindfulness therapy is a general term for a series of psychological training methods based on the theory of mindfulness, aimed at cultivating an individual’s ability to be aware of internal and external experience changes at the moment. Its effects have been verified in the auxiliary rehabilitation of chronic diseases, emotional disorders, and body pain (Wielgosz et al., 2019; Segal et al., 2020). It can effectively relieve anxiety and depression in different groups of people and improve sleep quality and quality of life. Additionally, neuroimaging studies have shown that mind-body awareness training (MBAT) can cause extensive reorganizations of the brain’s neural networks, particularly in the default-mode network (DMN), frontoparietal network (FPN) and limbic network (LN). By chance, crucial areas of the brain that are engaged in various aspects of BCI learning and neurofeedback mostly coincide with regions associated with meditation (Sitaram et al., 2017; Jiang H. et al., 2020). Meanwhile, previous trials have shown that mindfulness meditation can improve the effectiveness of BCIs in controlling emotions, promoting mental health, and reducing the risk of depression (Daudén Roquet and Sas, 2021). Neurophysiology also found that mindfulness therapy can enhance the activity of frontal limbic α (the regulation of frontal limbic α activity is positively correlated with meditation time and the improvement of BCI performance), and enhance the effectiveness of BCI control learning by preventing or reducing attention lapses (Jiang et al., 2021).

Thus, while BCI training system clearly improves motor function to stroke patients, the feasibility of using mindfulness therapy to improve BCI system accuracy in people with stroke hemiplegic patients has not been evaluated. That is, accelerating the rehabilitation process of stroke hemiplegic patients through the synergistic interaction between the motor and emotional systems. In this randomized clinical trial, we organically combined (i) BCI training system, (ii) mindfulness therapy, and (iii) rehabilitation care for stroke patients with hemiplegia, while relying on the BCI to decode the patient’s brain waves to induce active rehabilitation of the paralyzed limb Compared with participants receiving traditional rehabilitation training, we hypothesized that participants in the intervention group (receiving BCI and mindfulness therapy) would show a better improvement in physical health in terms of limb motor function and ctivities of daily living, mental health level of mindfulness and Sleep quality, and Quality of life.

## 2. Methods

### 2.1. Study design and participants

This study was a matched, evaluator-blind, randomized controlled trial conducted in China and has been registered with the Chinese Clinical Trial Registry (ChiCTR2300070382). A total of 128 eligible participants were recruited from the local Hospital from December 2021 to November 2022. All participants, including their legal guardian, provided written informed consent. The detailed design can be viewed through CONSORT checklist and CONSORT flow diagram (Schulz et al., 2010; Moher et al., 2012).

**FIGURE 1:**
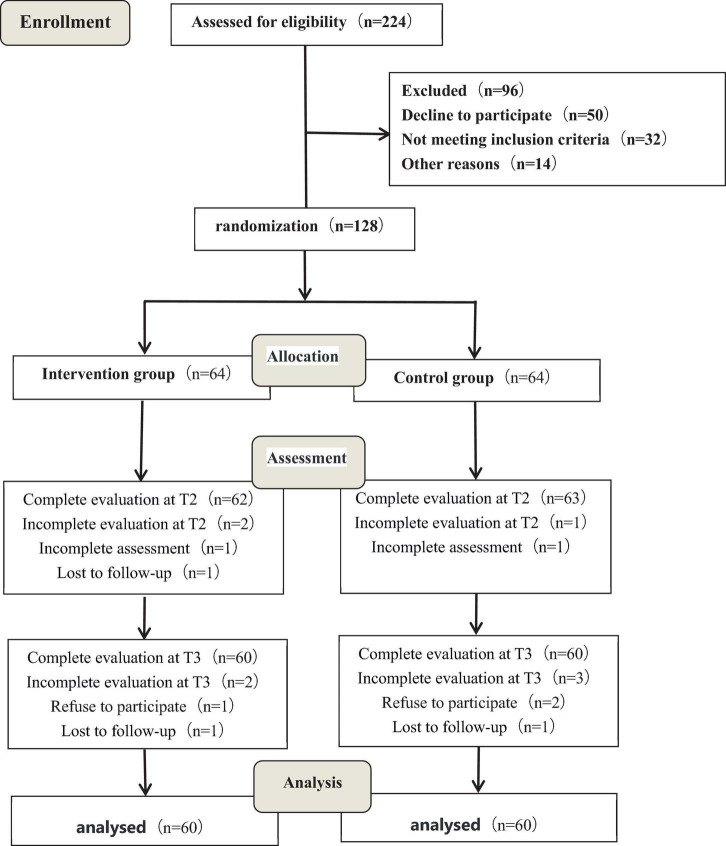
Enrollment of randomization.

The G*Power 3.1 program was used to calculate the sample size, and the test level was set at α = 0.05. Fifty patients in each group were required to ensure sufficient power (1-β = 0.9) to reject the null hypothesis. Considering a 20% loss rate during follow-up, at least 64 participants were required to be included in each group, and the total sample size in two groups was determined to be 128. The participants were randomly divided into an intervention group (*n* = 64) and a control group (*n* = 64) following a simple randomization procedure (computer-generated random numbers) (Figure 1).

The following were the inclusion criteria: (1) The patient was at least 18 years old; (2) patients diagnosed with stroke based on diagnostic criteria in accordance with the consensus on Clinical Research standards for acute Stroke in China formulated in 2018; (3) those that had experienced stroke for the first time, as confirmed by head CT or MRI (Table 1); (4) patients with unilateral hemiplegia, 4 weeks ≥ course ≤ 6 months, stable condition; and (5) those with moderate or severe limb dysfunction (limb Brunnstrom grade ≤ 4).

**TABLE 1:** The related to imaging studies.

Imageological examination	Method
CT (Computed Tomography)	It uses precisely collimated X-ray beams and highly sensitive detectors to scan a section of the human body after another. It has a quick scan time, clear images, and other advantages that make it useful for examining a wide range of diseases.
MRI (Magnetic Resonance Imaging)	It mainly uses a strong external magnetic field and hydrogen nuclei in the human body to produce magnetic resonance phenomenon when a specific radio frequency pulse is applied, and scans the diseased part of the patient to obtain the image of the part.

The exclusion criteria included the following: (1) patients with other nervous system diseases or severe heart, lung, and other important organ dysfunction or failure; (2) patients with a history of mental disorders and cognitive impairment (MMSE ≤ 21); (3) patients with visual impairment, hearing impairment, and history of taking psychotropic drugs in the past 6 months; (4) patients with metal implants, pacemakers, or skull defects; and (5) patients allergic to conductive glue.

### 2.2. Procedures

All participants received conventional rehabilitation therapy, including drug therapy, manual therapy, occupational therapy, acupuncture, moxibustion, neuromuscular electrical stimulation, and psychological counseling. The intervention group additionally received BCI training and mindfulness therapy after grouping using random number table method by nurses. This study wasn’t participant-blinded and intervener-blinded due to the concentration of the inpatient training site.

#### 2.2.1. Brain-computer interface training intervention

**FIGURE 2:**
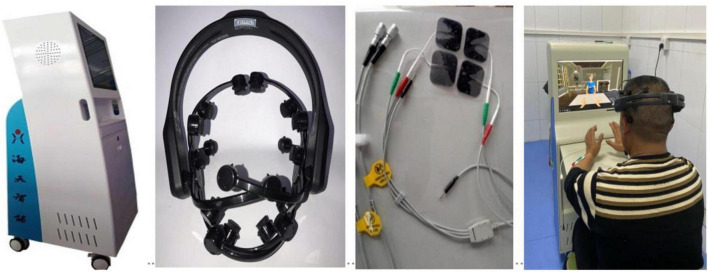
The brain-computer interface rehabilitation training system.

In the study, the BCI rehabilitation training system that was used was developed by Shandong Haitian Intelligent Engineering Co., Ltd. The BCI rehabilitation training system consisted of a host computer, EEG controller, electrical stimulation line, and BCI system software (Figure 2).

**FIGURE 3:**
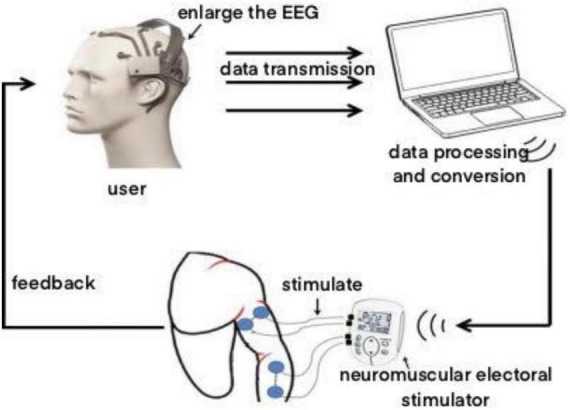
Working principle.

The working principle of brain-computer interface rehabilitation training system is that it integrates physical therapy and motor imagination therapy. Virtual reality scenarios are utilized to instruct stroke patients with hemiplegia to conduct repeated motor imagining, according to the rehabilitation training activities offered by the brain-computer interface rehabilitation training system. Then the non-invasive EEG collector gathers the patient’s EEG signal, processes the consciousness produced by the patient’s brain for particular actions, simulates the brain conduction pathway, evaluates the patient’s movement intention, operates the neuromuscular electrostimulation device to stimulate the nerves and muscles of the paralyzed limb, and supports and encourages the patient’s hemiplegic limb to complete the rehabilitation tasks prescribed by the doula. Furthermore, accelerate the restoration of damaged brain nerves and the formation of central conduction pathways. The working principle is shown in Figure 3.

The treatment was performed by researcher in rehabilitation center with a quiet, warm, and comfortable environment. Subsequently, the patient was assisted in seating, wearing an EEG collector, and adjusting the positions of the electrodes. After the EEG was successfully connected to the computer, the patient was instructed to perform motor imagery tasks with the hemiplegic limb according to language, action, and other prompts in the virtual reality animation. Meanwhile, EEG preprocessing, feature extraction, and pattern recognition were performed on the collected EEG signals to achieve timely feedback regulation for the participants; when the system estimated that the motor imagery task on the affected side was successful, it would automatically drive the biofeedback rehabilitation training instrument to induce the patient to produce an actual movement. During treatment, necessary communication was made with the patients, and the treatment experience, progress, expected efficacy, and other information were provided at any time in the process to increase patient compliance. The treatment duration was 20 min per day, 5 days a week for 8 weeks. The rehabilitation software supports the output result curve, and the result curve and the original EEG data will be saved for later analysis and diagnosis. It can also be uploaded to a cloud-data processing center for real-time display, storage, monitoring, and feedback. (In order to further verify the validity of the output results of the system, the original EEG signals collected synchronously during the treatment phase were analyzed offline. The results of offline classification are basically consistent with the results of online output of the rehabilitation training system, that is, above 77.5% of the imagined motion states correctly identified by the system are within the correct range of the algorithm).

#### 2.2.2. Mindfulness therapy intervention

The intervention time was performed once per week, for 80 min each time, and it was advanced synchronously with BCI training. The mindfulness therapy intervention program is shown in Table 2.

**TABLE 2:** Mindfulness therapy intervention program.

Stage	Time	Intervention content	Theme
First stage	Week 1–2	1. Before training, the participants will receive manuals related to mindfulness-based stress reduction (MBSR), which will let patients know the effect of rehabilitation training on hemiplegia, and introduce the purpose, content, key points and precautions of MBSR. Establish a good relationship with patients and keep them engaged.	Understand the significance of mindfulness for disease recovery, and gradually integrate attention into mindful breathing and other training.
		2. Guide the patient to primary training of MBSR, which includes mindful breathing, mindful listening, and mindful sitting meditation.	
		3. Give informal homework: 15 min mindful breathing training were performed (five times a week or more).	
Second stage	Week 3–4	1. Body scanning exercises, introducing walking meditation to patients, instructing them to systematically perceive each area of the body and consciously relax each muscle, generally starting from the toe to relax from the bottom up, and finally focusing on the whole body.	Constantly improve their ability to focus and experience themselves in different emotional states.
		2. Mindful eating raisins, through slowly chewing and swallowing, seriously understand the mindful eating brings to the body feeling.	
		3. Give informal homework: 10 min walking meditation training and 10 min mindful eating training were performed (five times a week or more).	
Third stage	Week 5–6	1. Mindfulness exercise training, consciously perceiving the thoughts and mental activities generated during active physical movement.	Accept and experience every thought, emotion from training, and try to live with them without insisting on change.
		2. Mindful mental scan, observe themselves inner changes at this moment from the perspective as an observer.	
		3. Give informal homework: 15 min mindfulness exercise training and 10 min mindfulness mental scan were performed (five times a week or more).	
Fourth stage	Week 7–8	Consolidate and practice exercises learned before, help patients change their understanding of diseases or negative emotions, develop the habit of looking at things with mindful thinking, encourage patients to accept themselves naturally to promote physical and mental health.	Start a new life of mindfulness, reunderstanding themselves and themselves illness.

### 2.3. Quality control

(1) All intervenators received training to thoroughly comprehend the study’s design, purpose, methodology, and tools, standardize the intervention process before the study, minimize the occurrence of false positive occurrences as much as feasible. (2) The researchers might collect the data by asking questions from the individuals who were unable to complete the questionnaire on their own. After the questionnaire was completed, each item was examined individually to identify any issues and have them quickly fixed, as well as to confirm the validity of the questionnaire, questionnaires with data missing > 10% were excluded. (3) To ensure that the allocation strategy was hidden during the trial, an independent researcher was in charge of creating the random number sequence, performing randomization, and putting the random numbers in sealed envelopes. (4) This subject has been approved by the Medical Research Ethics Committee of Shandong First Medical University. During the intervention, respondents had the right to refuse to participate and withdraw from the study at any time.

### 2.4. Outcome measures

The outcome assessors were blinded to group allocation and received standardized training. Participants were invited to a separate room in the hospital department for testing at three time points: before the intervention (T0), 3 months after the intervention (T1), and 6 months after the intervention (T2), to ensure that participants were not disturbed.

The primary outcome, limb motor function in stroke patients was measured by Fugl-Meyer assessment (FMA), which includes 8 items in the upper limbs (including wrist and hand functions) and 6 items in the lower limbs (including reflexes, hip, knee, ankle, etc.). Total score is 100 points, a high score suggests a high level of limb motor function. The FMA scale has good reliability and validity and is widely recognized internationally.

The secondary outcomes included (1) The activities of daily living was measured by Modified Barthel Index (MBI), which was derived from the revision of Barthel Index (BI) (Shah et al., 1989). It can objectively reflect the changes of activities of daily living in stroke patients with hemiplegia through 10 aspects (eating, bathing, grooming, dressing, defecation, urination, toileting, transferring, walking and going up and down stairs). (2) Participants’ levels of mindfulness, which was measured by Mindful Attention Awareness Scale (MAAS), it can reflect the individual’s ability to be aware of and pay attention to the present in daily life in a limited time. (3) The sleep quality, which was measured by Pittsburgh Sleep Quality Index (PSQI), was used to assess the sleep status of study subjects in the past month. The Cronbach’s α coefficient of the PSQI Chinese version scale is 0.842, which has good reliability and validity, and is a commonly used scale for the sleep study in China. (4) World Health Organization quality of life assessment instrument brief version (WHOQOL-BREF) was uesd to measure participants’ quality of life, it consists of four dimensions: physical, psychological, social and environmental, and two separate questions on general health and quality of life, this scale has now been widely used in stroke population.

### 2.5. Statistical analysis

All statistical analyses were performed by SPSS (version 25.0, IBM Corp.). All data were tested for normality. Count data including Gender, Age, Marital status, Education, Family monthly income, Medical expense, Etiology, Course of disease were shown as frequency (*n*) and percentage (%), and measurement data including FMA, ADL, MAAS, PSQI and WHOQOL-BREF were shown as mean ± standard deviation (M ± SD); *t*-test and χ^2^ test were used to compare the baseline between the two groups for general demographic data, disease-related data, activities of daily living, limb motor function, and secondary outcome indicators. The generalized estimating equation (GEE) was used to analyze the effects on limb motor function, activity of daily living, and secondary outcome indices at different treatment time points. The patients’ limb motor function, activities of daily living, mindfulness attention awareness level, sleep quality, and quality of life were measured at three time points: before the intervention (T0), 3 months after training (T1), and 3 months after training. *P* ≤ 0.05 (two-tailed) was set to indicate statistical significance. The trend graph of each outcome variable over time was plotted using GraphPad Prism software (version 9.4, GraphPad software Corp).

## 3. Results

### 3.1. Baseline demographics of the study population

A total of 128 patients were randomly divided into the intervention group (*n* = 64) or control group (*n* = 64). During the fieldwork, five participants refused to continue and three participants were uncontactable. Of the total number of participants who underwent randomization, 120 completed the 6-month intervention (intervention group, *n* = 60; control group, *n* = 60). The trial ended in March 2022, after all the intended data were collected. No obvious adverse injury was found during the study.

The baseline demographics of the study population appear in Table 3. The mean age of hemiplegic patients with stroke was 77.74 ± 7.44 years. Majority of participants had suffered from a cerebral infarction (72.5%), and over half of the participants had been ill for less than a month (52.5%). There was no statistically significant difference between the two groups (*P* > 0.05).

**TABLE 3:** The baseline demographics of the study population.

	Characteristics	Intervention (*n* = 60) *n* (%)	Control (*n* = 60) *n* (%)	χ^2^	*P*
Gender	Male	47 (78.3)	45 (75)	0.666	0.669
	Female	13 (21.7)	15 (25)		
Age (years)	≤ 45	6 (10)	3 (5)	0.530	0.641
	45–65	49 (81.7)	53 (88.3)		
	≥ 65	5 (8.3)	4 (6.7)		
Marital status	Married	59 (98.3)	59 (98.3)	1	1
	Unmarried or divorced	1 (1.7)	1 (1.7)		
Education	Primary school and below	43 (71.7)	39 (65)	0.546	0.678
	High/secondary school	11 (18.3)	16 (26.7)		
	Junior college and above	6 (10)	5 (8.3)		
Family monthly income	<2,000	10 (16.7)	12 (20)	0.888	0.775
(RMP)	2,000–5,000	37 (61.7)	35 (58.3)		
	≥ 5,000	13 (21.7)	13 (21.7)		
Medical expense	Self-funded	4 (6.7)	4 (6.7)	0.931	0.765
	Resident medical insurance	29 (48.3)	31 (51.7)		
	Employee medical insurance	27 (45)	25 (41.7)		
Etiology	Cerebral infarction	43 (71.7)	44 (73.3)	0.838	0.840
	Cerebral hemorrhage	17 (28.3)	16 (26.7)		
Course of disease	<1	32 (53.3)	31 (51.7)	0.855	0.720
(Month)	2–6	28 (46.7)	29 (48.3)		

### 3.2. Limb motor function

**FIGURE 4:**
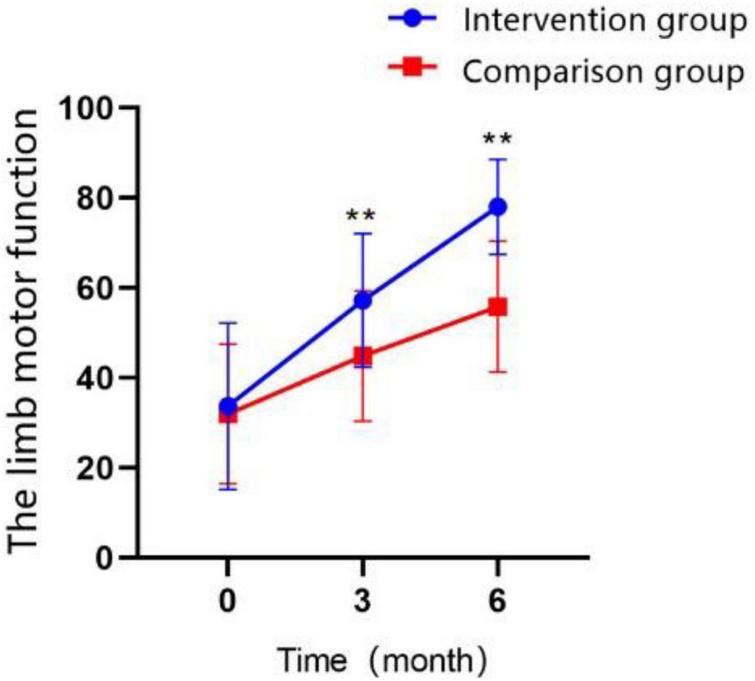
Changes in the scores of limb motor function over time in the intervention group and the control group (*P < 0.05, **P < 0.01).

Limb motor function was measured by the Fugl-Meyer Assessment (FMA), and the results demonstrated a significant improve with the extension of treatment time in both groups (Figure 4 and Table 4), but the intervention group always performed better than control group in T1 (MD = 12.32 ± 2.656, Cohen’s *d* = 0.83, *P* < 0.01) and T2 time points (MD = 22.20 ± 2.298, Cohen’s *d* = 1.74, *P* < 0.01). The interaction effect between the study groups and time was significantly different between the groups, as shown in the GEE (Table 5; *P* < 0.01).

**TABLE 4:** Descriptive statistics of patient scores at each time point and for each variable.

Variables	Time	M ± SD		*P*
		**Intervention**	**Control**	
The limb motor function	T0	33.70 ± 18.470	31.98 ± 15.514	0.582
	T1	57.20 ± 14.830	44.88 ± 14.514	<0.001
	T2	78.00 ± 10.519	55.80 ± 14.547	<0.001
Activity of daily living	T0	60.98 ± 16.611	64.05 ± 16.506	0.312
	T1	77.55 ± 9.668	72.62 ± 12.113	<0.05
	T2	88.03 ± 5.743	82.08 ± 8.327	<0.001
Mindful attention awareness	T0	48.62 ± 10.294	47.10 ± 10.787	0.432
	T1	67.93 ± 10.272	59.28 ± 9.546	<0.001
	T2	85.08 ± 7.089	73.77 ± 6.929	<0.001
Sleep quality	T0	15.18 ± 2.021	15.45 ± 1.826	0.450
	T1	12.08 ± 1.522	13.40 ± 1.689	<0.001
	T2	9.30 ± 1.169	11.02 ± 1.578	<0.001
The quality of life	T0	40.87 ± 1.556	41.40 ± 1.758	0.081
	T1	54.07 ± 3.686	46.42 ± 4.883	<0.001
	T2	66.08 ± 5.318	55.23 ± 5.037	<0.001

**TABLE 5:** The generalized estimated equation model effect test.

Outcome	Wald χ^2^	df	*P*
**FMA**			
Group	12.833	1	<0.001
Time	470.669	2	<0.001
Group × Time	17.358	2	<0.001
**ADL**			
Group	1.013	1	0.314
Time	314.197	2	<0.001
Group × Time	23.289	2	<0.001
**Maas**			
Group	15.112	1	<0.001
Time	1145.908	2	<0.001
Group × Time	38.181	2	<0.001
**PSQI**			
Group	19.021	1	<0.001
Time	3235.521	2	<0.001
Group × Time	123.573	2	<0.001
**WHOQOL-BREF**			
Group	183.041	1	<0.001
Time	2361.152	2	<0.001
Group × Time	186.137	2	<0.001

### 3.3. Activities of daily living

**FIGURE 5:**
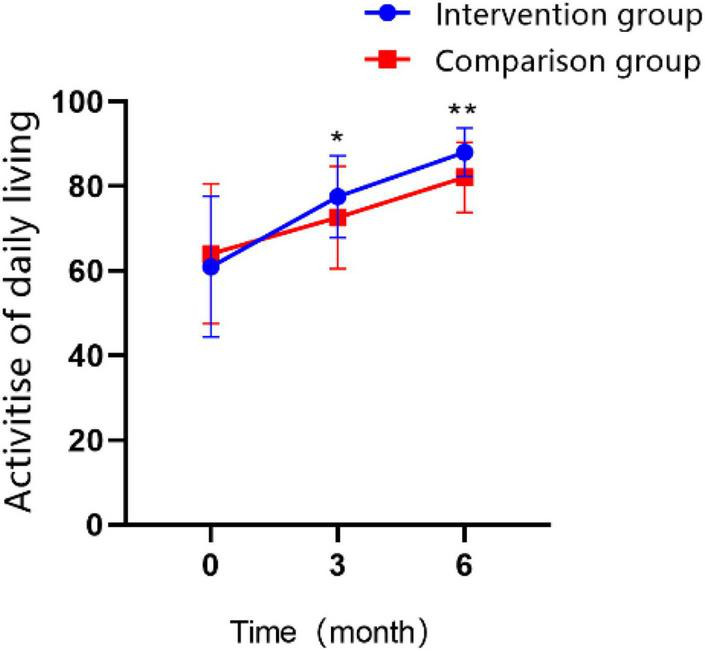
Changes in the scores of activity of daily living over time in the intervention group and the control group (*P < 0.05, **P < 0.01).

The participants’ activities of daily living were measured by the modified Barthel index (MBI), as time went on, improvement was observed in both groups: participants’ scores at T1 were higher than those at T0 in both groups, and the scores at T2 were higher than those at T0 and T1 stage for both groups (Figure 5; all *P* < 0.01). Comparing the two groups over the same time period, we found the differences were not statistically significant in MBI score between the two groups at 3 months of treatment, and patients in the intervention group recovered to a greater extent than those in control group after 6 months (MD = 5.95 ± 1.295, Cohen’s *d* = 0.83, *P* < 0.01). The interaction effect between the study groups and time was significantly different, as shown in the GEE analysis (Table 5; *P* < 0.01).

### 3.4. Mindful attention awareness

**FIGURE 6:**
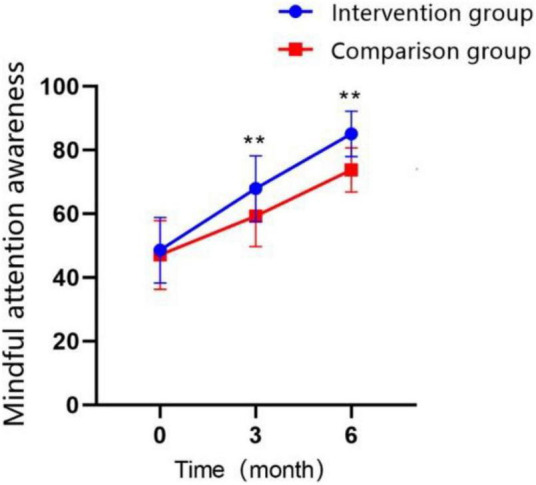
Changes in the scores of mindful attention awareness over time in the intervention group and the control group (*P < 0.05, **P < 0.01).

Participants’ mindful attention awareness was measured by mindful attention awareness scale (MAAS), demonstrated a significant increase with the extension of treatment time in both groups (Figure 6; *P* < 0.01). The MAAS scores in the intervention group were significantly higher than control group at T1 time point (MD = 8.65 ± 1.795, Cohen’s *d* = 0.87, *P* < 0.01), and the difference between two groups was even more significant after 3 months of continued treatment (MD = 11.30 ± 1.269, Cohen’s *d* = 1.61, *P* < 0.01). The GEE analysis revealed that the interaction effect between the study groups and time was significantly different (Table 5; *P* < 0.01).

### 3.5. Sleep quality

**FIGURE 7:**
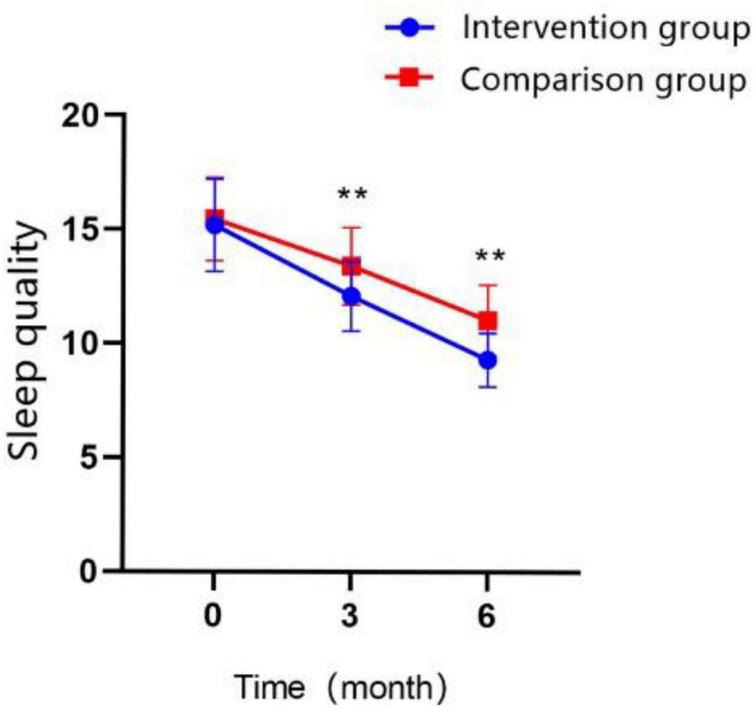
Changes in the scores of sleep quality over time in the intervention group and the control group (*P < 0.05, **P < 0.01).

In terms of PSQI scores on the follow-up test, participants in both groups improved their sleep quality significantly over time; the scores of PSQI in T1 were higher than those at T0 in both groups, and the total score at T2 was higher than those at T0 and T1 in both groups (Figure 7; all *P* < 0.01). The PSQI total score was significantly lower in the intervention group compared with that in the control group at T1 (MD = −1.32 ± 0.291, Cohen’s *d* = 0.82, *P* < 0.01) and at T2 (MD = −1.72 ± 0.254, Cohen’s *d* = 1.23, *P* < 0.01). At T2, most scores on the questionnaire subscales were lower than those of the control group, except for the sleep quality subscale. The GEE analysis revealed that the interaction effect between the study groups and time was significantly different (Table 5; *P* < 0.01).

### 3.6. Quality of life

**FIGURE 8:**
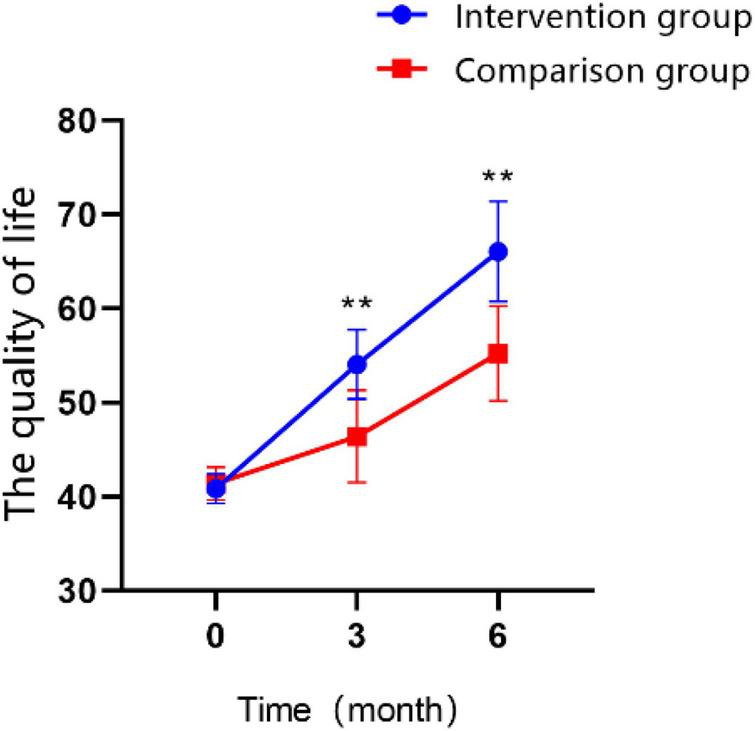
Changes in the scores of quality of life over time in the intervention group and the control group (*P < 0.05, **P < 0.01).

According to the assessment with the WHOQOL-BREF, an generic Quality of Life Scale developed by the World Health Organization, the quality of life improved significantly for both groups of patients was observed in the T1 and T2 time points (Figure 8; *P* < 0.01). Participants who received BCI combination with mindfulness therapy had a significantly better quality of life than the control group at T1 (MD = 7.65 ± 0.783, Cohen’s *d* = 1.76, *P* < 0.01) and at T2 (MD = 10.85 ± 0.938, Cohen’s *d* = 2.09, *P* < 0.01). The interaction effect between the study groups and time was significantly different in the GEE analysis (Table 5; *P* < 0.01).

## 4. Discussion

### 4.1. BCI combined with mindfulness therapy training can improve limb motor function, activities of daily living

In this randomized controlled trial, we evaluated the efficacy of BCI training combined with mindfulness therapy in 128 stroke patients. After 8 weeks of BCI combined with mindfulness intervention, this study showed that both groups had significant improvements in limb motor function and activities of daily living with extended rehabilitation time. Limb movement disorders, the most common post-stroke complication, are a major problem in clinical management owing to individual differences and the complexity of rehabilitation. In particular, upper limb motor function is an important predictor of return to normal work and life (Micera et al., 2020). Therefore, impairment of limb motor function can significantly reduce the ability to effectively exercises and activities of daily living, thereby reducing patients’ overall quality of life.

In the intervention group, compared to the control group, the limb motor function was better maintained at 3 and 6 months after the intervention, and the activities of daily living were better maintained at 6 months after the intervention. In addition, the changes in limb motor function and activities of daily living over time differed between the two groups, and the increasing trend (or trend of improvement) in the intervention group was observed to a greater extent than that in the control group. Several studies’ results are consistent with those of the present study. A meta-analysis (Mansour et al., 2022) included 298 post-stroke patients, showed that BCIs were more effective than traditional treatments in improving upper extremity/limb function (FMA-UE) in both subacute and chronic stroke patients. Another meta-analysis (Bai et al., 2020) showed that neuromuscular electrical stimulation triggered by a BCI significantly improved motor function and activities of daily living in patients with hemiplegia after stroke, and its long-term efficacy was verified during the follow-up period. The basis of BCI is neurofeedback training. When the patient performs the motor imagination task, the EEG electrical signal is analyzed by an external computer. When the brain engagement value of the motor image reaches a predetermined level, the external device supports the patient in completing the grasping movement of the disabled hand. The audiovisual stimulation motivates the patient to produce motor EEG signals, which cause the generation of new audiovisual stimuli, and so on, controlling the patient’s brain activity. When the patient completes the motor imagining task, the rehabilitation training system sends visual and motor feedback to the patient, therefore enhancing the operant conditioning reflex and achieving motor relearning. When the unilateral limb performs actual or imagined movement, the contralateral cerebral cortex region is activated, and the amplitude of motor evoked potential in this region increases accordingly, causing activation of the cortex related to motor planning in the pre-motor and auxiliary motor areas, and promoting cerebral cortex remodeling (Mrachacz-Kersting et al., 2016; Biasiucci et al., 2018; Moran and O’Shea, 2020). BCI technology allows the damaged brain to connect with external devices, re-establishing the relationship between motor-related cortical activities and proprioceptive feedback, strengthening the sensorimotor circuit, and promoting compensation and repair of damaged neurons.

Meanwhile, mindfulness intervention can significantly improve the performance of brain-computer interface training. Studies have shown that mindfulness meditation helps to focus attention, inhibit mind wandering, and overcome the interference of external things, indirectly affecting attention by changing the biological stress response related to emotions, and its neurophysiological mechanism is through the effective regulation of the amygdala. This was associated with decreased cortisol (a sympathetic nervous system marker of stress response) and improved vagus nerves (a marker of parasympathetic nervous system activation and relaxation). Therefore, mindfulness meditation can improve the task performance accuracy of BCI by reducing inattention (Lakey et al., 2011). A randomized controlled study (Schmid et al., 2014) evaluated the impact of 8 weeks of mindfulness and yoga practice on post-stroke physical function (range of motion, limb strength, and endurance); their findings revealed that compared to other patients, patients who received mindfulness combined yoga training showed a significant improvement in upper limb strength and walking speed. It has been suggested that BCI combined with mindfulness therapy can promote patients’ recovery of limb motor function. The possible reasons may be that in the process of mindfulness therapy, patients fully accept the discomfort and changes brought about by the disease and face, experience, and accept all their emotions and physical feelings with a positive attitude, which improves their control over emotions and their level of mindfulness. To maintain a stable mental state, patients can generate consistent and reliable EEG patterns when receiving BCI training, enhance EEG signals in the contralateral brain area to perform motor imagery tasks better, and beneficial to the recovery of affected limb function and physical and mental health.

### 4.2. BCI combined with mindfulness therapy training can improve mindful attention awareness

There was a two-way relationship between stroke occurrence and psychological factors. As a key factor in the development of stroke, psychological stress, severe self-perceived stress, stressful life events, and poor adaptability to stress are independently associated with increased risk of stroke (Saban et al., 2022). Physical dysfunction increases the psychological burden on patients, resulting in decreased activity endurance, excessive self-perceived burden, anxiety, depression, and other negative emotions, which seriously affect their health and quality of life. Our study showed that the level of mindful attention perception in the two groups gradually improved with the extension of rehabilitation time. At 3 and 6 months after the intervention, the observation, description, non-criticality, and non-reactivity to the internal experience of the intervention group were higher than those of the control group. In addition, the trend of the mindful attention perception level over time was different between the two groups, and the upward trend of the intervention group was greater than control group. A randomized controlled trial (Cladder-Micus et al., 2018) was conducted in 106 patients with chronic, refractory depression by Cladder-Micus et al. (2018) showed that mindfulness cognitive therapy was effective in reducing the risk of depression recurrence. A meta-analysis systematically evaluated the effects of mindfulness training on rehabilitation of patients after stroke and found that mindfulness training could effectively improve the sensorimotor function of the limbs after stroke (Zou et al., 2018). It has been suggested that BCI training combined with mindfulness therapy can improve the awareness of mindful attention in stroke patients with hemiplegia. This may be because positive thinking provides a simple and effective way to help patients focus their attention to the present, openly, consciously, and continuously; they are not constrained by personal likes, dislikes, opinions, or prejudices, thus enabling them to escape trouble and return to regular life. A longitudinal study (Stieger et al., 2021) found that mindfulness therapy increased neurological alpha band (conscious resting state) activity levels, thereby enhancing the effectiveness of BCI-controlled learning. Jiang X. et al. (2020) explored the effect of long-term mindfulness meditation on the learning of BCIs based on sensorimotor rhythms. The behavioral and neurophysiological differences between experienced mindfulness meditators and controls were analyzed. The results showed that long-term meditation experience could affect the average performance of the sensorimotor rhythm-based BCI, sensorimotor rhythm-based predictor, resting-state mu stability, and control signals during task execution. Therefore, mindfulness therapy can stimulate the cerebral cortex to control attention and emotion, thereby improving the patient’s compliance with BCI rehabilitation training, completion of motor imagery tasks, and strengthening the patient’s sense of self-control.

### 4.3. BCI combined with mindfulness therapy training can improve sleep quality and quality of life

Post-stroke patients are more likely to experience sleep disorders that are linked to post-stroke depression. When sleep quality declines, depressive symptoms become more severe. Negative feelings not only interfere with recovery but also increase the chance of impairment and significantly lower patients’ quality of life. This study showed that sleep quality and quality of life in two groups of patients gradually improved with the extension of rehabilitation time. The total scores of sleep quality, quality of life, and the scores of each dimension of the intervention group were better than those of the control group at 3 months and 6 months after the intervention. The trends of sleep quality and quality of life over time were different between the two groups, and the upward trend of the intervention group was greater than that of the control group. Bower et al. (2021) reported a randomized controlled study, which also explored the effect of psychosomatic intervention (mindfulness meditation training) on sleep quality in breast cancer survivors. A total of 85 patients who received standardized mindfulness meditation training were compared with 81 patients who received conventional sleep education. The results is consistent with our study: the sleep quality of the intervention group was significantly improved, and the levels of fatigue and depression were significantly reduced. On the one hand, BCI training combined with mindfulness therapy guides patients to carry out regular motor imagery rehabilitation exercises; on the other hand, it cultivates patients to use mindfulness thinking to view the disease in a positive light, promotes patients’ physical and mental relaxation, effectively controls self-behavior, and allows them to perform functional exercise in a better state, thereby improving the effect of rehabilitation training and making it more conducive to improve sleep and quality of life.

## 5. Study limitations

This study had certain limitations. First, non-invasive BCIs still have limitations in the processing of EEG signals, and it remains a challenge to accurately decode the freedom of arm and leg movement, control, and grasp different objects. Second, the BCI training system used in this study has an imperfect exercise prescription and a single exercise mode, which cannot accurately match the exercise prescription to the actual rehabilitation needs of patients. Third, the benefits of BCI training combined with mindfulness therapy in stroke patients need to be explored in an overall mechanistic model, including electrophysiological, hemodynamic, and neurochemical components. Finally, our results might have been influenced by the fact that intervention group participants spent more time using BCI than control group participants. In the future, it will be necessary to simultaneously measure neurological changes related to the BCI combined with mindfulness therapy in different modalities.

## 6. Conclusion

In conclusion, we confirmed that Brain–Computer Interface training combined with mindfulness therapy can improve limb motor function, activities of daily living, mindful attention awareness, sleep quality, and quality of life in patients with hemiplegia after stroke. Our findings provide a theoretical and practical basis for rehabilitation interventions for stroke patients with hemiplegia.

## Data availability statement

The raw data supporting the conclusions of this article will be made available by the authors, without undue reservation.

## Ethics statement

The studies involving humans were approved by the Medical Ethics Committee of Shandong First Medical University. The studies were conducted in accordance with the local legislation and institutional requirements. The participants provided their written informed consent to participate in this study. Written informed consent was obtained from the individual(s) for the publication of any potentially identifiable images or data included in this article.

## Author contributions

PW responsible for the intervention and data collection. JL mainly responsible for the data collection and writing manuscript. AZ provided the statistical guidance and thesis guidance. LW guided the clinical intervention and writing of thesis. HM and XM revised the guidance for manuscript return. All authors contributed to the article and approved the submitted version.

## References

[B1] Bai Z. Fong K. N. K. Zhang J. J. Chan J. Ting K. H. (2020). Immediate and long-term effects of BCI-based rehabilitation of the upper extremity after stroke: A systematic review and meta-analysis. *J. Neuroeng. Rehabil.* 17:57. 10.1186/s12984-020-00686-2 32334608 PMC7183617

[B2] Baillieul S. Dekkers M. Brill A. K. Schmidt M. H. Detante O. Pépin J. L. (2022). Sleep apnoea and ischaemic stroke: Current knowledge and future directions. *Lancet Neurol.* 21 78–88. 10.1016/S1474-4422(21)00321-5 34942140

[B3] Biasiucci A. Leeb R. Iturrate I. Perdikis S. Al-Khodairy A. Corbet T. (2018). Brain-actuated functional electrical stimulation elicits lasting arm motor recovery after stroke. *Nat. Commun.* 9:2421. 10.1038/s41467-018-04673-z 29925890 PMC6010454

[B4] Bower J. E. Partridge A. H. Wolff A. C. Thorner E. D. Irwin M. R. Joffe H. (2021). Targeting depressive symptoms in younger breast cancer survivors: The pathways to wellness randomized controlled trial of mindfulness meditation and survivorship education. *J. Clin. Oncol.* 39 3473–3484. 10.1200/JCO.21.00279 34406839 PMC8547916

[B5] Bright F. A. Kayes N. M. Worrall L. Mcpherson K. M. (2015). A conceptual review of engagement in healthcare and rehabilitation. *Disabil. Rehabil.* 37 643–654. 24969698 10.3109/09638288.2014.933899

[B6] Cervera M. A. Soekadar S. R. Ushiba J. Millán J. D. R. Liu M. Birbaumer N. (2018). Brain-computer interfaces for post-stroke motor rehabilitation: A meta-analysis. *Ann. Clin. Transl. Neurol.* 5 651–663. 29761128 10.1002/acn3.544 PMC5945970

[B7] Chaudhary U. Mrachacz-Kersting N. Birbaumer N. (2021). Neuropsychological and neurophysiological aspects of brain-computer-interface (BCI) control in paralysis. *J. Physiol.* 599 2351–2359. 10.1113/JP278775 32045022

[B8] Cladder-Micus M. B. Speckens A. E. M. Vrijsen J. N. Ar T. D. Becker E. S. Spijker J. (2018). Mindfulness-based cognitive therapy for patients with chronic, treatment-resistant depression: A pragmatic randomized controlled trial. *Depress Anxiety* 35 914–924. 10.1002/da.22788 30088834 PMC6175087

[B9] Danzl M. M. Etter N. M. Andreatta R. D. Kitzman P. H. (2012). Facilitating neurorehabilitation through principles of engagement. *J. Allied Health* 41 35–41. 22544406

[B10] Daudén Roquet C. Sas C. (2021). A mindfulness-based brain-computer interface to augment mandala coloring for depression: Protocol for a single-case experimental design. *JMIR Res. Protoc.* 10:e20819. 10.2196/20819 33459604 PMC7850910

[B11] Feigin V. L. Stark B. A. Johnson C. O. Roth G. A. Bisignano C. Abady G. G. (2021). Global, regional, and national burden of stroke and its risk factors, 1990-2019: A systematic analysis for the global burden of disease study 2019. *Lancet Neurol.* 20 795–820. 34487721 10.1016/S1474-4422(21)00252-0 PMC8443449

[B12] Fotakopoulos G. Kotlia P. (2018). The Value of exercise rehabilitation program accompanied by experiential music for recovery of cognitive and motor skills in stroke patients. *J. Stroke Cerebrovasc. Dis.* 27 2932–2939. 10.1016/j.jstrokecerebrovasdis.2018.06.025 30072173

[B13] Gittler M. Davis A. M. (2018). Guidelines for adult stroke rehabilitation and recovery. *JAMA* 319 820–821. 29486016 10.1001/jama.2017.22036

[B14] Guo J. Wang J. Sun W. Liu X. (2022). The advances of post-stroke depression: 2021 update. *J. Neurol.* 269 1236–1249. 10.1007/s00415-021-10597-4 34052887

[B15] Hardwick R. M. Caspers S. Eickhoff S. B. Swinnen S. P. (2018). Neural correlates of action: Comparing meta-analyses of imagery, observation, and execution. *Neurosci. Biobehav. Rev.* 94 31–44. 30098990 10.1016/j.neubiorev.2018.08.003

[B16] Jiang H. He B. Guo X. Wang X. Guo M. Wang Z. (2020). Brain-heart interactions underlying traditional tibetan buddhist meditation. *Cereb. Cortex* 30 439–450. 10.1093/cercor/bhz095 31163086

[B17] Jiang H. Stieger J. Kreitzer M. J. Engel S. He B. (2021). Frontolimbic alpha activity tracks intentional rest BCI control improvement through mindfulness meditation. *Sci. Rep.* 11:6818. 10.1038/s41598-021-86215-0 33767254 PMC7994299

[B18] Jiang X. Lopez E. Stieger J. R. Greco C. M. He B. (2020). Effects of long-term meditation practices on sensorimotor rhythm-based brain-computer interface learning. *Front. Neurosci.* 14:584971. 10.3389/fnins.2020.584971 33551719 PMC7858648

[B19] Lakey C. E. Berry D. R. Sellers E. W. (2011). Manipulating attention via mindfulness induction improves P300-based brain-computer interface performance. *J. Neural Eng.* 8:025019. 10.1088/1741-2560/8/2/025019 21436516 PMC4429763

[B20] Malik A. N. Tariq H. Afridi A. Rathore F. A. (2022). Technological advancements in stroke rehabilitation. *J. Pak. Med. Assoc.* 72 1672–1674. 36280946 10.47391/JPMA.22-90

[B21] Mansour S. Ang K. K. Nair K. P. S. Phua K. S. Arvaneh M. (2022). Efficacy of brain-computer interface and the impact of its design characteristics on poststroke upper-limb rehabilitation: A systematic review and meta-analysis of randomized controlled trials. *Clin. EEG Neurosci.* 53 79–90. 10.1177/15500594211009065 33913351 PMC8619716

[B22] Micera S. Caleo M. Chisari C. Hummel F. C. Pedrocchi A. (2020). Advanced neurotechnologies for the restoration of motor function. *Neuron* 105 604–620. 32078796 10.1016/j.neuron.2020.01.039

[B23] Moher D. Hopewell S. Schulz K. F. Montori V. Gøtzsche P. C. Devereaux P. J. (2012). CONSORT 2010 explanation and elaboration: Updated guidelines for reporting parallel group randomised trials. *Int. J. Surg.* 10 28–55. 22036893 10.1016/j.ijsu.2011.10.001

[B24] Moran A. O’Shea H. (2020). Motor imagery practice and cognitive processes. *Front. Psychol.* 11:394. 10.3389/fpsyg.2020.00394 32194491 PMC7063062

[B25] Mrachacz-Kersting N. Jiang N. Stevenson A. J. Niazi I. K. Kostic V. Pavlovic A. (2016). Efficient neuroplasticity induction in chronic stroke patients by an associative brain-computer interface. *J. Neurophysiol.* 115 1410–1421. 10.1152/jn.00918.2015 26719088 PMC4808132

[B26] Owolabi M. O. Thrift A. G. Mahal A. Ishida M. Martins S. Johnson W. D. (2022). Primary stroke prevention worldwide: Translating evidence into action. *Lancet Public Health* 7 e74–e85. 34756176 10.1016/S2468-2667(21)00230-9 PMC8727355

[B27] Perera K. S. De Sa Boasquevisque D. Rao-Melacini P. Taylor A. Cheng A. Hankey G. J. (2022). Evaluating rates of recurrent ischemic stroke among young adults with embolic stroke of undetermined source: The young ESUS longitudinal cohort study. *JAMA Neurol.* 79 450–458. 10.1001/jamaneurol.2022.0048 35285869 PMC8922202

[B28] Pfurtscheller G. Neuper C. Guger C. Harkam W. Ramoser H. Schlögl A. (2000). Current trends in Graz brain-computer interface (BCI) research. *IEEE Trans. Rehabil. Eng.* 8 216–219. 10.1109/86.847821 10896192

[B29] Rost N. S. Brodtmann A. Pase M. P. Van Veluw S. J. Biffi A. Duering M. (2022). Post-stroke cognitive impairment and dementia. *Circ. Res.* 130 1252–1271. 35420911 10.1161/CIRCRESAHA.122.319951

[B30] Saban K. L. Tell D. De La Pena P. (2022). Nursing implications of mindfulness-informed interventions for stroke survivors and their families. *Stroke* 53 3485–3493. 10.1161/STROKEAHA.122.038457 35904017

[B31] Saunders D. H. Sanderson M. Hayes S. Johnson L. Kramer S. Carter D. D. (2020). Physical fitness training for stroke patients. *Cochrane Database Syst. Rev.* 3:Cd003316. 10.1002/14651858.CD003316.pub7 PMC7083515 32196635

[B32] Schmid A. A. Miller K. K. Van Puymbroeck M. Debaun-Sprague E. (2014). Yoga leads to multiple physical improvements after stroke, a pilot study. *Complement. Ther. Med.* 22 994–1000. 25453519 10.1016/j.ctim.2014.09.005

[B33] Schulz K. F. Altman D. G. Moher D. (2010). CONSORT 2010 statement: Updated guidelines for reporting parallel group randomised trials. *J. Pharmacol. Pharmacother.* 1 100–107. 21350618 10.4103/0976-500X.72352 PMC3043330

[B34] Segal Z. V. Dimidjian S. Beck A. Boggs J. M. Vanderkruik R. Metcalf C. A. (2020). Outcomes of online mindfulness-based cognitive therapy for patients with residual depressive symptoms: A randomized clinical trial. *JAMA Psychiatry* 77 563–573. 31995132 10.1001/jamapsychiatry.2019.4693 PMC6990961

[B35] Shah S. Vanclay F. Cooper B. (1989). Predicting discharge status at commencement of stroke rehabilitation. *Stroke* 20, 766–769. 10.1161/01.str.20.6.766 2728043

[B36] Sitaram R. Ros T. Stoeckel L. Haller S. Scharnowski F. Lewis-Peacock J. (2017). Closed-loop brain training: The science of neurofeedback. *Nat. Rev. Neurosci.* 18 86–100. 28003656 10.1038/nrn.2016.164

[B37] Stieger J. R. Engel S. Jiang H. Cline C. C. Kreitzer M. J. He B. (2021). Mindfulness improves brain-computer interface performance by increasing control over neural activity in the alpha band. *Cereb. Cortex* 31 426–438. 10.1093/cercor/bhaa234 32965471 PMC7727383

[B38] Wielgosz J. Goldberg S. B. Kral T. R. A. Dunne J. D. Davidson R. J. (2019). Mindfulness meditation and psychopathology. *Annu. Rev. Clin. Psychol.* 15 285–316. 30525995 10.1146/annurev-clinpsy-021815-093423 PMC6597263

[B39] Zou L. Sasaki J. E. Zeng N. Wang C. Sun L. (2018). A Systematic review with meta-analysis of mindful exercises on rehabilitative outcomes among poststroke patients. *Arch. Phys. Med. Rehabil.* 99 2355–2364. 10.1016/j.apmr.2018.04.010 29738744

